# Applicability, reliability, and accuracy of age‐at‐death estimation methods on a contemporary Italian population

**DOI:** 10.1111/1556-4029.70011

**Published:** 2025-03-13

**Authors:** Paolo Morandini, Lucie Biehler‐Gomez, Cristina Cattaneo

**Affiliations:** ^1^ Laboratorio di Antropologia e Odontologia Forense (LABANOF), Department of Biomedical Science for Health University of Milan Milan Italy

**Keywords:** acetabulum, aging methods, auricular surface, clavicle sternal end, first rib, forensic anthropology, older adults, palatine sutures, pubic symphysis, skeletal age estimation

## Abstract

This research tested the applicability, intra‐ and inter‐observer reliability, and accuracy of nine macroscopic methods for estimating age‐at‐death from skeletal elements. The sample included 400 individuals from the contemporary CAL Milano Cemetery Skeletal Collection, equally divided by sex assigned at birth and with age‐at‐death ranging from 20 to 104 years. Statistical analyses used standard measures of reliability and validity. The most applicable methods assessed the auricular surface of the ilium (92%), whereas the preservation and/or identification of the fourth rib was rare (16%). All methods proved repeatable, except for the assessment of the obliteration of palatine sutures, which showed greater subjectivity to the observer's experience. The Rougé‐Maillart (2009) method exhibited low bias and inaccuracy across all age groups in both sexes and the strongest correlation with chronological age in males. In females, the Buckberry and Chamberlain 2002 method showed the strongest correlation, but it tended to overestimate the age of younger individuals in both sexes. Age‐at‐death estimation methods revealed varying accuracy across age groups. The Suchey‐Brooks 1990 method was effective for younger individuals, while the Rougé‐Maillart (2009) and Falys and Prangle 2015 methods showed potential for the estimation of older adults, with lower bias and better precision. However, no approach was entirely satisfactory for older adults. The increasing life expectancy and the likelihood of encountering older adults’ remains highlight the need for refined techniques that better address age diversity in contemporary and ancient populations.

**Highlights**

- Applicability, reliability, and accuracy testing of nine skeletal age estimation methods.
- Rougé‐Maillart (2009) exhibited the least inaccuracy and bias across age groups.
- Palatine sutures showed the worst reliability, accuracy, and correlation with age.
- Falys and Prangle (2015) showed potential for the age estimation of older adults.
- Validation studies are essential to test the validity of methods in specific populations.

## 1 INTRODUCTION

Estimating age‐at‐death from skeletal remains is a crucial step in constructing the biological profile, essential in both forensic contexts, for the identification of unknown individuals [1], and bioarchaeological studies, to assess demographic profiles of past populations [2]. A fundamental assumption underlying age‐at‐death estimation from skeletal remains is that a strong correlation exists between chronological and biological ages, allowing biological age‐at‐death estimation to serve as a predictor for chronological age [3, 4]. However, biological aging is shaped by a complex interplay of genetic, cultural, and environmental factors, meaning that a perfect match between chronological and biological ages does not necessarily exist [5, 6]. Health status, diet, and individual activity are all factors capable of altering the rate of growth and aging in various tissues, including bone, thereby influencing an individual's biological age [3]. The influence of these extrinsic factors varies between individuals and across different populations, and at any given chronological age, biological age may differ among individuals within a population and between populations [7]. Moreover, as chronological age progresses, the cumulative impact of these extrinsic factors further differentiates biological age from actual age over time [3]. Consequently, age‐at‐death estimation in adults is less precise than in juveniles, with even the best methods providing age‐at‐death estimates with a range of 10–15 years [8]. Age‐at‐death estimation becomes even more challenging for older individuals, where current methods lack the precision to determine narrow age‐at‐death ranges [8, 9]; hence, age intervals for older individuals are often limited to a broad and insufficient “50+” category [10].

Traditionally, age‐at‐death estimation in adults using macroscopic skeletal analysis focuses on four primary anatomical regions: cranial sutures, the pubic symphysis, the auricular surface, and the sternal end of the fourth rib. Studies aimed at finding methods to estimate age‐at‐death from these skeletal structures began as early as the 1800s, with the studies of Paul Broca and Paul Topinard [11, 12]. Since then, numerous methods examining these traits have been developed, reevaluated, and updated using different samples, and many original methods remain popular and widely used [13]. Consequently, more recent studies have focused on exploring other anatomical areas to develop increasingly precise and accurate methods.

The pubic symphysis remains the most widely relied upon area for age‐at‐death estimation, with the Suchey‐Brooks method [14] remaining the preferred technique [13]. This predilection stems from the method's basis on a large, diverse sample, its detailed morphological descriptions, and extensive testing on various collections [e.g., 15, 16, 17, 18, 19].

The use of the auricular surface for age‐at‐death estimation was first proposed in Lovejoy et al.'s publication [20], which is still very popular [13]. The auricular surface is favored because it is anatomically resistant to taphonomic changes, and its aging process appears unaffected by sex, biogeographic origin, or secular changes [21]. Buckberry and Chamberlain [22] revised the Lovejoy 1985 method, replacing the staging approach with a numerical scoring system. The authors' goal was to make the technique easier to apply and thus reduce intra‐ and interobserver error. A subsequent validation study on the Terry and Huntington Collections found that the method applies equally well to population affinities of both White and Black individuals of both sexes, but younger individuals (20–49 years) are less accurately estimated compared to the original Lovejoy 1985 method [23]. More recently, Osborne et al. [21] provided updated age ranges for auricular surface analysis, while Hens and Godde [24] introduced a Bayesian approach using transition analysis statistics to refine age estimates for modern American skeletal samples. These studies highlight ongoing efforts to improve the accuracy and applicability of auricular surface‐based age estimation methods. A recent survey found that the Lovejoy 1985 method remains the most applied for auricular surface age estimation, though the Buckberry and Chamberlain 2002 method is also used, albeit less frequently, while newer methods are rarely applied [13].

Another anatomical area under consideration, especially alongside the auricular surface, is the acetabulum. This area is one of the most taphonomically resistant and shows a slow process of growth, which correlates to age [25]. While some authors focused solely on the acetabulum [26, 27, 28, 29], Rougé‐Maillart et al. [30] developed a new method based on the combined evaluation of acetabular and auricular surface degeneration. The method proved reliable and accurate, particularly in providing precise classification even for older individuals, including those over 60 [30]. A subsequent test on a known Italian sample confirmed the method's potential for estimating age in the older adults, allowing for a more precise and accurate classification compared to traditional methods [9].

The sternal end of the fourth rib is also among the main anatomical areas of interest, with the Iscan method [31, 32] still being commonly used [13]. In forensic settings, the Işcan method is straightforward to apply: opening the thoracic cavity during autopsy allows examination of the fourth rib and, if needed, its collection and maceration [4]. Additionally, the ability to visualize morphological changes through medical imaging, such as X‐rays or 3D tomography, broadens the method's applicability to fresh cadavers [33]. However, its application is limited in bioarchaeological contexts due to the difficulty of complete recovery and identification [9]. Although the Işcan method was originally developed for the sternal end of the right fourth rib, Yoder et al. [34] tested its applicability to other ribs (II, III, V–XI), finding significant differences only between the second and fourth ribs. However, they advised caution when applying the method to other ribs due to concerns regarding statistical significance.

Recently, the focus has also extended to evaluating the degeneration of the first rib, particularly following the work of Kunos et al. in 1999 [35]. In their publication, the authors critique the Iscan method for its limited applicability in disarticulated skeletons. They argue that rib‐based age estimation is incomplete if it only relies on the sternal end, as it overlooks other aspects that undergo age‐related changes, and that the first rib is more easily identifiable and likely to be well preserved [35, 36]. Consequently, Kunos et al. [35] developed a method for age‐at‐death estimation that combines three aspects of the first rib: the sternal end, head, and tubercle. However, subsequent validation tests of the method have highlighted certain limitations, including the fact that the methodology is difficult to apply, time intensive, and not always accurate [37, 38]. Specifically, unlike the validation test proposed by Kunos [35], findings revealed that individuals aged over 60 were often misclassified [38].

In addition to cranial vault sutures, palatal suture closure has also been investigated. The first method was proposed by Mann in 1987 [39], later revised in 1991 [40]. Two subsequent validation tests produced conflicting results: Ginter [41] reported the method to be accurate for estimating the age of older individuals, whereas Gruspier and Mullen [42] observed significant variability in age prediction, recommending against its use. In 2010, Beauthier and colleagues proposed a different approach, dividing the sutures into 15 regions with five obliteration stages to derive obliteration coefficients and regression equations [43]. The authors believed in its potential for older individuals, for whom commonly used methods often lose precision [43]. A recent study on a limited Italian sample confirmed that the method was useful for estimating ages in the range of 60–80 years but underestimated those older than 80 [9]. More recently, Hens and Godde [44] applied Bayesian statistics to palatine sutures, introducing a modified scoring system that separates the transverse palatine suture into two distinct locations. Their study tested both transition analysis and Bayesian multiple linear regression, demonstrating that the latter produced narrower and more accurate age ranges while minimizing bias [44].

In recent years, the sternal end of the clavicle has been examined for adult age estimation. The clavicle is the last bone in the human skeleton to complete maturation, with the sternal end typically fusing between the late second and early third decades of life [45, 46]. Consequently, evaluating the fusion degree of this anatomical portion has proven useful for estimating age in younger adults [47, 48]. In 2015, Falys and Prangle [49] published the first method based on the sternal end of the clavicle after fusion, applicable to both middle‐aged and older adults. Initial validation tests demonstrated a strong correlation between the clavicle's morphological features and age‐at‐death [50, 51]. The method appears to have high accuracy rates, with moderate inter‐observer agreement [52] and proved applicable and useful in cremated skeletal remains [53]. However, the method was developed on individuals aged 40 years and older, failing to identify individuals within the 25–40 age range [50, 51, 52].

The accuracy of a method can vary depending on several factors, one of which is the so‐called age mimicry bias. The concept of age mimicry was introduced by Bocquet‐Appel and Masset [54] within paleodemography and has since been methodologically explained by various authors [55, 56, 57]. Fundamentally, this bias causes estimates of the target population to mimic the age demographic structure of the reference population. As a result, age estimates may reflect the demographic and biological characteristics of the reference individuals rather than accurately representing the age of the individual in question. A statistical approach that has proven effective in addressing this issue is transition analysis [10, 18, 58]. Originally developed by Boldsen et al. [58], transitional analysis is a probabilistic method that utilizes binary or ordinal reference data to generate more statistically robust age estimates. Unlike traditional techniques, transitional analysis incorporates multiple skeletal traits—such as cranial sutures and pelvic joints—while accounting for correlations between them and integrating information about population mortality structure. Furthermore, literature indicates that no single method provides accurate estimates for the entire human lifespan and different age estimation methods yield more accurate results for certain age groups [59]. Thus, it is also important to consider whether certain methods tend to overestimate younger adults or underestimate older ones [13]. Although interpopulation variation in age‐at‐death estimation characteristics is well established, there is debate in the literature about how much this affects the final accuracy of estimates. Some authors argue that population differences are not necessarily significant enough to alter the results of these methods, suggesting that instead of focusing on variations between ethnic groups, efforts should be made to obtain larger and more diverse baseline samples [60, 61]. However, a prevailing idea in the literature is that the closer the reference sample is to the study case, the more accurate the estimate will be [13]. Consequently, it is important to conduct validation tests for methods across different populations to assess their validity for specific groups [62].

Regarding the Italian population, some methods have been tested on contemporary known samples. In 2008, Hens et al. [62] tested the Suchey‐Brooks 1990 and Lovejoy 1985 methods on a sample of 404 individuals from the osteological collection of Sassari, preserved at the Museum of Anthropology of the University of Bologna. The results indicated that the methods perform well up to the age of 40, beyond which age tends to be underestimated. Moreover, degeneration of the auricular surface showed a better correlation with the actual age than the pubic symphysis, with lower levels of bias and inaccuracy even for individuals over 40, especially males [62]. In 2012, Hens and Belcastro tested the Buckberry and Chamberlain 2002 method on the same sample, showing better accuracy than the original Lovejoy 1985 method, although individuals under 60 tended to be overestimated, while those over 60 were underestimated [63]. Cappella et al. [9] used a sample of 145 individuals from the CAL Milano Cemetery Skeletal Collection to test the applicability and accuracy of five methods: Suchey‐Brooks [14], Lovejoy [20], Rougé‐Maillart [30], Iscan [31, 32], and Beauthier [43]. The sample mainly included individuals over 60 years of age to evaluate the validity of different methods for older individuals. However, as acknowledged by the authors, the sample was limited in number and, being confined to a specific age range, did not allow a comprehensive overview of the validity of the methods. Furthermore, the application of the Beauthier 2010 and Iscan 1984–1985 methods was limited to just over 20% of the sample due to taphonomic factors, greatly restricting the validity of findings regarding the accuracy of these methods [9]. In contrast, and to the best of our knowledge, other methods such as Kunos [35] or the more recent Falys and Prangle [49] have not been tested on an Italian known sample.

In this context, this research aims to test (1) the applicability, (2) the reliability, and (3) the accuracy of the most commonly used macroscopic methods for adult age‐at‐death estimation on a contemporary Italian sample. Specifically, the research seeks to identify the strengths and limitations of each method, determine which age groups yield the highest accuracies and suggest methods that may be more effective for addressing age estimation challenges in older adults. The results will also offer practical guidance for improving the application of these methods in both forensic and bioarchaeological contexts.

## 2 MATERIALS AND METHODS

**FIGURE 1:**
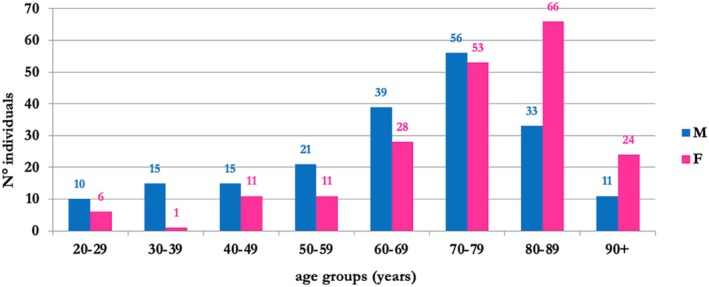
Sample distribution by sex assigned at birth and age group.

The sample included 400 skeletons, drawn from the CAL Milano Cemetery Skeletal Collection, housed within the Laboratory of Forensic Anthropology and Odontology (LABANOF) of the Department of Biomedical Sciences for Health of the University of Milan. This contemporary and documented collection consists of unclaimed remains from Milan cemeteries, obtained under Article 43 of the Mortuary Police Regulation (DPR No. 285, September 10th, 1990) [64]. Additional documentation, including known sex assigned at birth and age‐at‐death, was provided for each individual. The selected sample was evenly split between sexes (200 males and 200 females). Age‐at‐death ranged from 20 to 104 years, with an average age‐at‐death of 66 years for males (Standard Deviation—SD = 18; range: 20–101) and 75 years for females (SD = 16; range: 21–104), with birth years from 1880 to 1972 and death years spanning from 1927 to 2001. The sample was divided into eight age groups (Figure 1). The number of individuals within each age group is not evenly distributed, as it reflects the mortality pattern of the contemporary Milanese population. Consequently, the most represented age groups are the older adults, with 77.5% of the sample over 60 years.

Nine macroscopic methods for adult age‐at‐death estimation were tested (Table 1): the Lovejoy 1985 (LO85) [20] and the Buckberry and Chamberlain 2002 (BC02) [22] methods for the auricular surface of the ilium; the Rougé‐Maillart (2009) (RM09) [30] method, combining auricular surface and acetabulum; the Suchey‐Brooks 1990 (SB90) [14] method for the pubic symphysis; the Iscan (1984 for males and 1985 for females) (IS84‐85) [31, 32] methods concerning the sternal end of the fourth rib; the Kunos 1999 (KU99) [35] method for the first rib; the Beauthier 2010 (BE10) [43] and Mann 1991 (MA91) [40] methods for the degree of obliteration of the palatine sutures; and the Falys and Prangle 2015 (FP15) [49] method for the degeneration of the sternal end of the clavicle. These methods were selected based on their prevalence in the literature, with the inclusion of more recent methods such as Rougé‐Maillart (2009) and Falys and Prangle (2015), which, although less extensively tested, have demonstrated potential, particularly for estimating age‐at‐death in older adults.

**TABLE 1:** Summary of the methodologies applied in this study.

Method	Abbreviation	Description	Methodology	References
Suchey‐Brooks 1990	SB90	Each pubic symphysis was assigned to one of six phases, with each phase corresponding to an age range defined as the mean age ± standard deviation, as outlined in the methodology guidelines	Phases with age ranges	[14]
Lovejoy 1985	LO85	Each auricular surface was classified into one of eight stages, each linked to a specific age range. For bias and inaccuracy calculations, the midpoint of the range was used as the estimated age. For the final stage (>60 years), an estimated age of 65 was adopted, consistent with Hens et al. (2008) [62]	Stages with age ranges	[20]
Buckberry and Chamberlain 2002	BC02	Each auricular surface was scored according to the criteria specified in the original publication. The estimated age and standard deviation of the composite score groups were then used for age‐at‐death estimation	Composite scores with age ranges	[22]
Iscan 1984–1985	IS84/IS85	Each sternal end of the fourth rib was classified into one of the nine proposed phases and the respective estimated age and standard deviation were calculated separately for male (IS84) and female (IS85) samples	Phases with age ranges	[31, 32]
Kunos 1999	KU99	Each of the three features of the method (costal end, tubercle, head) was assessed, with the oldest observable description selected for each characteristic. The respective age was considered the minimum age‐at‐death, providing an age range defined only by a minimum value. For the final estimation, the oldest age observed among the three features was used. Bias and inaccuracy were calculated based on this final estimation as the estimated age	Stages descriptions with minimum age‐at‐death	[35]
Beauthier 2010	BE10	Palatine sutures were scored following the guidelines of the method. Sex‐specific regression equations were applied (age estimated for females = (7.4 × IN) − (0.5 × AMP) + (1.84 × TP) + (0.79 × PMP) − 77.8; age estimated for males = (5.94 × IN) − (1.53 × AMP) + (2.37 × TP) + (3.86 × PMP) − 81.9), providing a point estimate for age. To create an age range, a margin of ±10 years was added to the estimate.	Regression equations	[43]
Mann 1991	MA91	To avoid ambiguity in quantifying the degree of obliteration of the sutures, the approach suggested by Beauthier (2010) [43] was followed, considering “complete obliteration” when the suture showed more than 75% obliteration. Based on these assessments, each individual was classified into one of the age groups indicated. For bias and inaccuracy calculations, the midpoint of the range was used as the estimated age. For the final stage (>50 years), an estimated age of 50 was adopted to calculate bias and inaccuracy	Stages with age ranges	[40]
Rougé‐Maillart (2009)	RM09	A composite score was calculated for each innominate bone. For this study, the most probable age range associated with the composite score groups was taken as one standard deviation, while other acceptable age ranges were considered for two standard deviations. For bias and inaccuracy calculations, the midpoint of the range was used as the estimated age, while, for the final stage (>84 years), an estimated age of 84 was adopted	Composite scores with age ranges	[30]
Falys and Prangle 2015	FP15	Each sternal end of the clavicle was scored following the guidelines of the method. Both the composite score system and the regression equations provided by the method were evaluated. Regarding the composite score system, the mean ages, standard deviations, and 95% confidence intervals for the score groups were used. For the regression formulas, the sex‐specific equations were applied, along with the standard deviations specified by the method	Composite scores with age ranges/Regression equations	[49]

These methods were applied to each skeleton when the anatomical region of interest was preserved and assessable. The analyses were conducted blind to assigned sex at birth and age‐at‐death; however, the scoring of different anatomical features was not performed independently. The tests were conducted by the original investigator of the paper (PM), who had prior experience in applying these methodologies. When both sides were available, the side exhibiting the most aged appearance was chosen, as this approach has yielded the most accurate results in the literature, at least concerning the pubic symphysis [65]. For intra‐observer analysis, 17 individuals were randomly selected, and the estimations were repeated by the same observer (PM) approximately 6 months later. For inter‐observer analysis, 15 skeletons were randomly selected, and estimations were performed by two observers (PM and LB‐G): Observer 1 (PM) had 3 years of prior experience in applying these methods on archaeological skeletal remains, while Observer 2 had a decade of experience and had directly taught Observer 1.

Descriptive statistics and subsequent analyses were performed using Microsoft Excel® and JASP® software (version 0.18.3). For both intra‐ and inter‐observer reliability, Cohen's unweighted Kappa was computed [66]. The interpretation of the results followed the guidelines available in the literature [67].

An independent samples *t*‐test was performed to assess differences in age distribution between male and female samples. The assumption of equal variances was verified using Levene's test, confirming that standard *t*‐test assumptions were met. Accuracy was assessed through standard measures of bias and inaccuracy used in similar studies [e.g., 60, 66]: Bias represents the mean over‐ or underestimation, calculated as Σ(estimated age − actual age)/*n*, whereas inaccuracy is the mean absolute error of age estimation, without regard to over‐ or underestimation, expressed as Σ|estimated age − actual age|/*n* (where *n* represents the number of observations). Additionally, the percentage of correctness of each method was calculated, defined as the proportion of correctly estimated individuals (C%), whose actual ages fell within the estimated age range, relative to the total number of individuals observed. Conversely, if the estimated age range exceeded the actual age, the individual was classified as overestimated (O%), and if the estimated age range was lower, as underestimated (U%). Furthermore, the correlation between chronological age‐at‐death and estimated age‐at‐death for each method was computed using Pearson's correlation coefficient for parametric data and Spearman's correlation coefficient for non‐parametric data.

## 3 RESULTS

### 3.1 Applicability

Applicability data are summarized in Table 2. The number varies considerably depending on the anatomical area of interest. The most applicable methods were those assessing the auricular surface of the ilium: LO85 (368; 92%), BC02 (368; 92%), and RM09, which also includes evaluation of the acetabular portion (361; 90.3%). The pubic symphysis was preserved in just over half of the cases, allowing for the SB90 method in 233 individuals (58.3%). In contrast, preservation (and/or identification) of the fourth rib was rare, limiting the application of the IS84–85 method to only 64 individuals (16%).

**TABLE 2:** Number of individuals tested and percentage of applicability of each method based on the survival of their relative skeletal elements.

Method	M	F	T	M%	F%	T%
Lovejoy 1985	184	184	368	92.0%	92.0%	92.0%
Buckberry e Chamberlain 2002	184	184	368	92.0%	92.0%	92.0%
Rougé‐Maillart (2009)	182	179	361	91.0%	89.5%	90.3%
Falys e Prangle 2015	152	132	284	76.0%	66.0%	71.0%
Kunos 1999	147	135	282	73.5%	67.5%	70.5%
Beauthier 2010	144	124	268	72.0%	62.0%	67.0%
Mann 1991	144	124	268	72.0%	62.0%	67.0%
Suchey‐Brooks 1990	130	103	233	65.0%	51.5%	58.3%
Iscan 1984–1985	38	26	64	19.0%	13.0%	16.0%

Abbreviations: F, number of females; F%, percentage in the female sample; M, number of males; M%, percentage in the male sample; T, total number; T%, percentage in the total sample.

### 3.2 Intra‐ and inter‐observer analysis

Table 3 summarizes the results of the unweighted Cohen's Kappa. The results demonstrate high levels of repeatability, with intra‐ and inter‐observer agreement ranging from “substantial” to “almost perfect” for most features and composite scores. An exception is the BE10 method, which showed lower reliability than the other techniques. Specifically, in the intra‐observer analysis, the anterior medial palatine (AMP) suture indicated only “fair” agreement between the two repetitions, while in the inter‐observer analysis, the scoring of palatine sutures reached only “slight” or “moderate” agreement.

**TABLE 3:** Summary of intra‐ and interobserver rate agreement for each method.

Method	Feature	Intra‐observer			Inter‐observer		
		*n*	*K*	Agreement	*n*	*K*	Agreement
Rougé‐Maillart (2009)	Transverse organization	17	0.852	*Almost perfect*	13	0.594	*Moderate*
	Surface texture	17	0.794	*Substantial*	13	0.606	*Substantial*
	Porosity	17	0.920	*Almost perfect*	13	0.889	*Almost perfect*
	Apical activity	17	0.891	*Almost perfect*	13	1.000	*Perfect*
	Rim	17	0.913	*Almost perfect*	13	0.785	*Substantial*
	Fossa	17	1.000	*Perfect*	13	0.667	*Substantial*
	Apical activity posterior cornus	17	0.909	*Almost perfect*	13	0.737	*Substantial*
	Composite Score	17	0.804	*Almost perfect*	13	0.649	*Substantial*
Buckberry and Chamberlain 2002	Transverse organization	17	0.750	*Substantial*	13	0.649	*Substantial*
	Surface texture	17	0.897	*Almost perfect*	13	0.606	*Substantial*
	Microporosity	17	0.876	*Almost perfect*	13	0.794	*Substantial*
	Macroporosity	17	1.000	*Perfect*	13	0.764	*Substantial*
	Apical changes	17	1.000	*Perfect*	13	1.000	*Perfect*
	Composite Score	17	0.821	*Almost perfect*	13	0.698	*Substantial*
Lovejoy 1985	Stage	17	1.000	*Perfect*	13	0.870	*Almost perfect*
Suchey‐Brooks 1990	Phase	13	1.000	*Perfect*	11	1.000	*Perfect*
Iscan 1984–1985	Phase	7	1.000	*Perfect*	2	1.000	*Perfect*
Falys and Prangle 2015	Topography	16	0.842	*Almost perfect*	11	0.880	*Almost perfect*
	Porosity	16	1.000	*Perfect*	11	0.699	*Substantial*
	Osteophyte formation	16	1.000	*Perfect*	11	0.735	*Substantial*
	Composite Score	16	0.854	*Almost perfect*	11	0.780	*Substantial*
Beauthier 2010	IN	14	0.903	*Almost perfect*	11	0.427	*Moderate*
	AMP	14	0.349	*Fair*	11	0.193	*Slight*
	TP	14	0.528	*Moderate*	11	0.124	*Slight*
	PMP	14	0.803	*Almost perfect*	11	0.542	*Moderate*
Mann 1991	Age group	14	0.881	*Almost perfect*	11	0.625	*Substantial*
Kunos 1999	Costal face	14	0.804	*Almost perfect*	10	0.846	*Almost perfect*
	Tubercle	13	0.743	*Substantial*	8	0.692	*Substantial*
	Head	10	0.773	*Substantial*	12	0.782	*Substantial*

Abbreviations: AMP, anterior medial palatine suture; IN, incisive palatine suture; *K*, Cohen's unweighted Kappa; *n*, number of individuals tested; PMP, posterior median palatine suture; TP, Transverse palatine suture.

### 3.3 Accuracy

The two‐tailed independent samples *t*‐test indicated a statistically significant difference in age distribution between the male and female samples (*p* < 0.001). Consequently, the following analyses were conducted separately for each sex. Tables S1–S6 show the descriptive statistics for each method analyzed.

Table 4 summarizes the correlation coefficients obtained for each method. Among males, the composite score from RM09 showed the strongest correlation with age‐at‐death (*R* = 0.925), whereas, in the female sample, the highest association was found with the composite score from BC02 (*R* = 0.845). Conversely, the degree of palatal suture obliteration exhibited the weakest correlation with age‐at‐death for both sexes, with particularly low results in the female sample. Specifically, the degree of obliteration of the IN suture showed no significant correlation with age‐at‐death in females (*R* = 0.043; *p* = 0.633), nor did the AMP suture (*R* = 0.126; *p* = 0.163). Additionally, the age class indicated by the MA91 method did not show a statistically significant correlation with chronological age‐at‐death in the female sample (*R* = 0.157; *p* = 0.082).

**TABLE 4:** Results of the correlation coefficients (*R*) between features and chronological age for each method.

Methods	Features	Males		Females	
		*R*	*p*‐value	*R*	*p*‐value
Rougé‐Maillart (2009)	Transverse organization	0.796	<0.001	0.557	<0.001
	Surface Texture	0.742	<0.001	0.515	<0.001
	Porosity	0.704	<0.001	0.581	<0.001
	Apical activity	0.752	<0.001	0.585	<0.001
	Rim	0.785	<0.001	0.568	<0.001
	Fossa	0.576	<0.001	0.573	<0.001
	Apical activity posterior cornus	0.668	<0.001	0.532	<0.001
	Composite score	0.925	<0.001	0.819	<0.001
Buckberry and Chamberlain 2002	Transverse organization	0.713	<0.001	0.595	<0.001
	Surface texture	0.739	<0.001	0.753	<0.001
	Microporosity	0.672	<0.001	0.629	<0.001
	Macroporosity	0.565	<0.001	0.559	<0.001
	Apical changes	0.743	<0.001	0.702	<0.001
	Composite score	0.868	<0.001	0.845	<0.001
Lovejoy 1985	Stage	0.880	<0.001	0.808	<0.001
Suchey‐Brooks 1990	Phase	0.756	<0.001	0.753	<0.001
Iscan 1984–1985	Phase	0.677	<0.001	0.793	<0.001
Falys and Prangle 2015	Topography	0.677	<0.001	0.643	<0.001
	Porosity	0.781	<0.001	0.686	<0.001
	Osteophyte formation	0.685	<0.001	0.605	<0.001
	Composite score	0.791	<0.001	0.757	<0.001
Beauthier 2010	IN	0.333	<0.001	0.043	0.633*
	AMP	0.334	<0.001	0.126	0.163*
	TP	0.334	<0.001	0.308	<0.001
	PMP	0.438	<0.001	0.238	0.008
	Cp	0.420	<0.001	0.274	0.002
	Age (regression)	0.461	<0.001	0.204	0.023
Mann 1991	Age group	0.305	0.008	0.157	0.082*
Kunos 1999	Costal face	0.611	<0.001	0.706	<0.001
	Tubercle	0.635	<0.001	0.800	<0.001
	Head	0.583	<0.001	0.724	<0.001

*Note*: Cp = coefficient of obliteration of the palatine sutures (Beauthier et al. [43]).

* *p*‐Value not significant (*p* > 0.05).

Table 5 reports the percentages of individuals correctly estimated, overestimated, or underestimated by sex. Within one standard deviation, the male sample showed the highest accuracy rate with the LO85 method (79.3%), while for the female sample, the KU99 method performed best, with an accuracy of 84.4%. As illustrated in Table 6, the results of LO85 reveal further nuances when examining individuals above and below 60 years of age: For those over 60, the method achieved an 88.8% accuracy rate in correct classification, whereas for those under 60, the percentage dropped sharply, reaching only 56%.

**TABLE 5:** Results of the percentages of individuals correctly estimated (C%), overestimated (O%), or underestimated (U%) within one standard deviation by sex for each method.

Age group	SB90			LO85			BC02			RM09			IS85			KU99			BE10			MA91			FP15 cs			FP15 re		
	C%	O%	U%	C%	O%	U%	C%	O%	U%	C%	O%	U%	C%	O%	U%	C%	O%	U%	C%	O%	U%	C%	O%	U%	C%	O%	U%	C%	O%	U%
**Males**																														
20–29	83.3	16.7	0.0	33.3	66.7	0.0	88.9	11.1	0.0	77.8	22.2	0.0	100	0.0	0.0	33.3	66.7	–	0.0	60.0	40.0	20.0	0.0	80.0	0.0	100	0.0	83.3	16.7	0.0
30–39	77.8	22.2	0.0	53.3	40.0	6.7	46.7	53.3	0.0	64.3	35.7	7.1	100	0.0	0.0	10.0	90.0	–	46.2	23.1	30.8	0.0	23.1	76.9	0.0	100	0.0	69.2	30.8	0.0
40–49	85.7	0.0	14.3	76.9	7.7	15.4	30.8	69.2	0.0	61.5	23.1	7.7	66.7	16.7	16.7	25.0	75.0	–	50.0	16.7	33.3	0.0	16.7	83.3	46.2	53.8	0.0	53.8	46.2	0.0
50–59	92.9	0.0	7.1	78.9	0.0	21.1	63.2	31.6	5.3	78.9	10.5	10.5	75.0	25.0	0.0	26.7	73.3	–	27.8	27.8	44.4	33.3	0.0	66.7	71.4	28.6	0.0	71.4	21.4	7.1
60–69	89.3	0.0	10.7	70.3	0.0	29.7	100	0.0	0.0	43.2	27.0	29.7	100	0.0	0.00	60.7	39.3	–	56.3	12.5	31.3	34.4	0.0	65.6	62.1	34.5	3.4	62.1	20.7	17.2
70–79	33.3	0.0	66.7	89.1	0.0	10.9	100	0.0	0.0	54.5	27.3	18.2	38.5	0.0	61.5	93.2	6.8	–	42.9	14.3	42.9	34.3	0.0	65.7	93.0	4.7	2.3	55.8	2.3	41.9
80–89	0.0	0.0	100	96.4	0.0	3.6	75.0	0.0	25.0	59.3	7.4	33.3	0.0	0.0	100	100	0.0	–	34.8	0.0	65.2	17.4	0.0	82.6	58.3	0.0	41.7	50.0	0.0	50.0
90+	0.0	0.0	100	100	0.0	0.0	0.0	0.0	100	75.0	0.0	25.0	0.0	0.0	100	100	0.0	–	16.7	0.0	83.3	33.3	0.0	66.7	20.0	0.0	80.0	30.0	70.0	0.0
TOT	53.1	2.3	44.6	79.3	7.6	13.0	78.8	13.0	8.2	58.8	23.1	18.1	43.8	3.1	53.1	68.0	32.0	–	41.0	14.6	44.4	25.0	3.5	71.5	59.2	27.6	13.2	57.9	18.4	23.7
**Females**																														
20–29	100	0.0	0.0	50.0	50.0	0.0	66.7	33.3	0.0	50.0	50.0	0.0	33.3	0.0	66.7	75.0	25.0	–	20.0	60.0	20.0	40.0	0.0	60.0	–	–	–	–	–	–
30–39	0.0	0.0	100.0	0.0	100.0	0.0	0.0	100	0.0	100	0.0	0.0	–	–	–	–	–	–	0.0	0.0	100.0	0.0	0.0	100	0.0	100	0.0	100	0.0	0.0
40–49	100	0.0	0.0	20.0	30.0	50.0	50.0	50.0	0.0	80.0	10.0	10.0	80.0	0.0	20.0	44.4	55.6	–	55.6	22.2	22.2	0.0	0.0	100	22.2	77.8	0.0	66.7	33.3	0.0
50–59	100	0.0	0.0	60.0	30.0	10.0	50.0	40.0	10.0	60.0	20.0	20.0	0.0	0.0	100	55.6	44.4	–	42.9	0.0	57.1	0.0	0.0	100	80.0	10.0	10.0	100	0.0	0.0
60–69	70.0	0.0	30.0	76.0	0.0	24.0	100	0.0	0.0	24.0	28.0	48.0	33.3	0.0	66.7	68.4	31.6	–	27.8	5.6	66.7	5.6	0.0	94.4	80.0	15.0	5.0	95.0	0.0	5.0
70–79	28.0	0.0	72.0	88.0	0.0	12.0	100	0.0	0.0	55.3	29.8	14.9	33.3	0.0	66.7	85.7	14.3	–	18.9	0.0	81.1	16.2	0.0	83.8	85.3	14.7	0.0	70.6	0.0	29.4
80–89	0.0	0.0	100	96.8	0.0	3.2	53.2	0.0	46.8	68.9	1.6	29.5	0.0	0.0	100	100	0.0	–	5.7	0.0	94.3	17.1	0.0	82.9	56.8	11.4	31.8	61.4	0.0	38.6
90+	0.0	0.0	100	100	0.0	0.0	0.0	0.0	100	78.9	0.0	21.1	0.0	0.0	100	100	0.0	–	0.0	0.0	100	8.3	0.0	91.7	0.0	0.0	100	28.6	71.4	0.0
TOT	31.1	0.0	34.0	83.7	5.4	10.9	66.3	7.1	26.6	60.3	16.2	23.5	26.9	0.0	73.1	84.4	15.6	–	18.5	4.8	76.6	19.4	0.0	78.6	60.6	17.4	22.0	68.9	9.8	21.2

Abbreviations: cs, composite score approach of FP15; re, regression equation approach of the FP15.

**TABLE 6:** Accuracy of the Lovejoy 1985 method by sex and age groups (<60 years and > 60 years).

	<60 years				>60 years			
	*n*	C%	O%	U%	*n*	C%	O%	U%
M	56	64.3%	23.2%	12.5%	128	85.9%	–	14.1%
F	27	40.7%	37.0%	22.2%	157	91.1%	–	8.9%
TOT	83	56.6%	27.7%	15.7%	285	88.8%	–	11.2%

Abbreviations: C%, percentage of individuals correctly classified; *n*, number of individuals tested; O%, percentage of individuals overestimates; U%, percentage of individuals underestimated.

**FIGURE 2:**
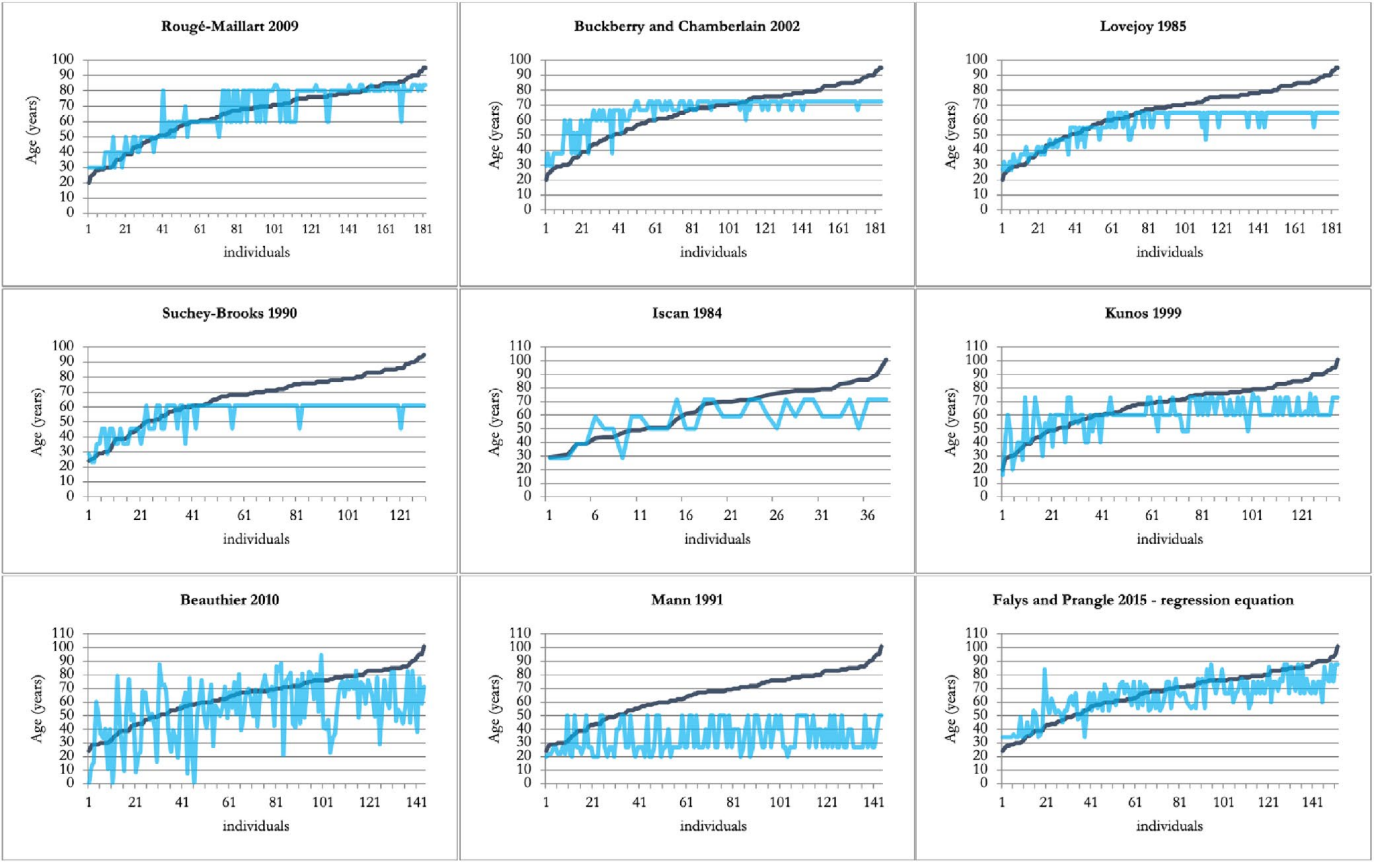
Comparison of known age‐at‐death (dark blue line) and estimated age‐at‐death (light blue line) for the male sample across all analyzed age‐at‐death estimation methods. Points falling above or below the line of known age indicate overestimation or underestimation, respectively.

**FIGURE 3:**
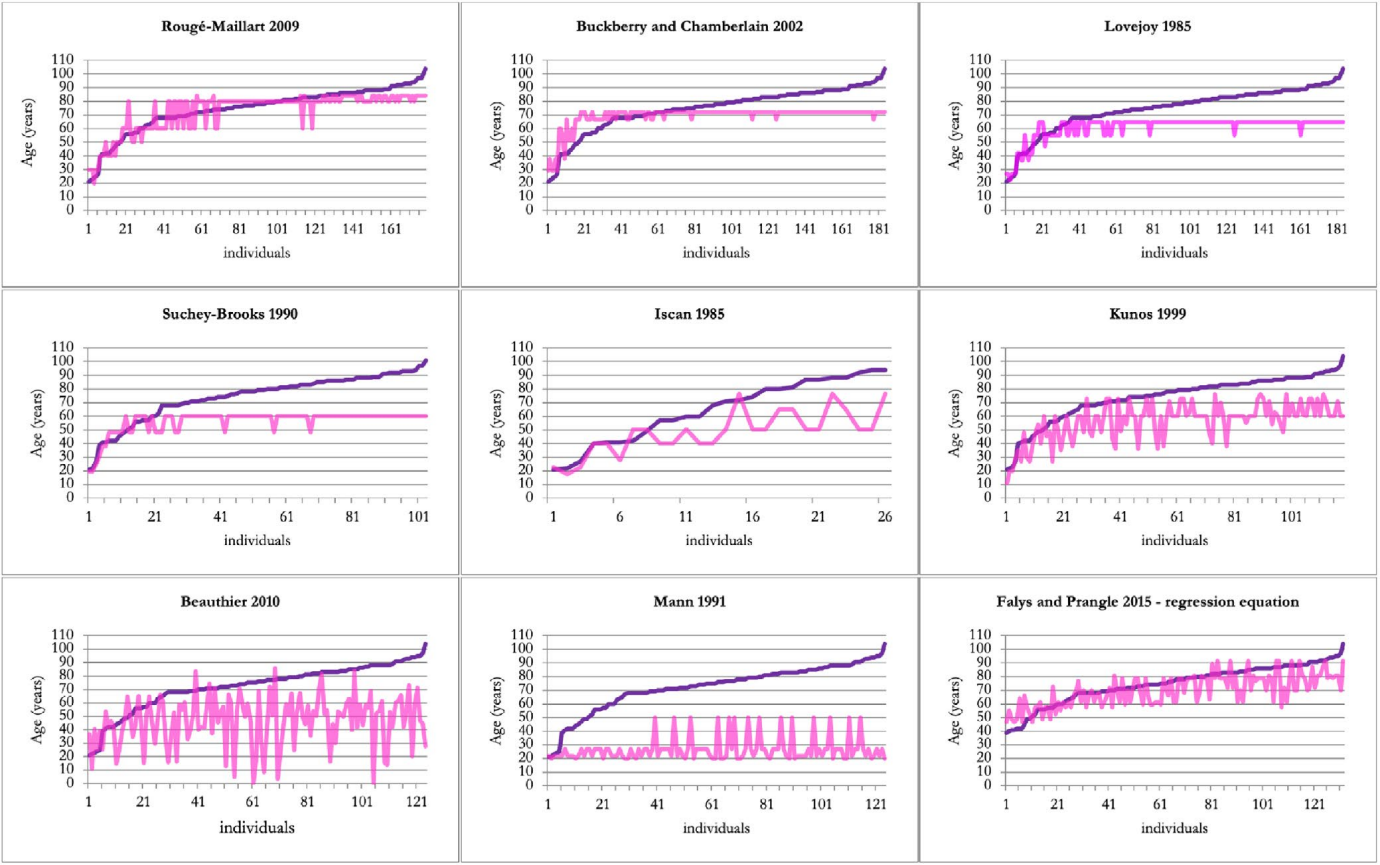
Comparison of known age‐at‐death (dark purple line) and estimated age‐at‐death (light purple line) for the female sample across all analyzed age‐at‐death estimation methods. Points falling above or below the line of known age indicate overestimation or underestimation, respectively.

Table 7 and Figures 2 and 3 summarize bias and inaccuracy results of the methods. In the female sample, all methods tended to underestimate the individuals' actual age. The composite score approach of FP15 showed the bias closest to zero (bias = −1.02), indicating a better balance between underestimation and overestimation, with a low total inaccuracy (7 years), second only to RM09 (6 years). For the male sample, RM09 reports the lowest bias (0.9) and the lowest total inaccuracy (5.8 years). Additionally, it is the only one analyzed that exhibited consistently low bias and inaccuracy across all age groups. To investigate further the RM09 results, the data collected was processed using the Bayesian method to obtain the conditional probabilities of each age category for the sample under study (Table 8). Based on Table 8, the following observations can be made:

- Only one individual was classified as **Group I**, a 24‐year‐old female.
- Within **Group II**, 94% of individuals were aged 20–34, with a maximum age of 36.
- Within **Group III**, 75% of individuals were in the 35–44 range, with a minimum age of 30 and a maximum age of 49.
- Within **Group IV**, there was a 62% probability of being in the 45–54 range; in males, individuals were aged 31–63; in females, 42–60.
- Within **Group V**, 90% of individuals were in the 55–74 age range, with an equal probability of belonging to either the 55–64 or 65–74 age groups.
- Within **Group VI**, 99% of individuals were over 65; only two individuals under 65 (one aged 51 and another one aged 56) were in this score group.
- Within **Group VII**, all individuals were at least 71 years old, and 78% were older than 84 years.

**TABLE 7:** Results of bias (B) and inaccuracy (I) by sex and age group for each method.

Age group	SB90			LO85			BC02			RM09			IS84‐85			KU99			BE10			MA91			FP15 cs			FP15 re		
	*n*	B	I	*n*	B	I	*n*	B	I	*n*	B	I	*n*	B	I	*n*	B	I	*n*	B	I	*n*	B	I	*n*	B	I	*n*	B	I
**Males**																														
20–29	6	5.1	6.5	9	3.9	4.1	9	8.6	8.6	9	4.0	4.0	1	−0.8	0.8	6	9.3	9.3	5	−2.4	23.4	5	−4.2	4.2	6	21.5	21.5	6	7.3	7.3
30–39	9	3.5	7.0	15	3.1	3.7	15	14.6	14.7	14	4.4	5.6	4	−1.3	1.3	10	8.3	10.6	13	2.3	18.5	13	−4.7	11.5	13	14.9	14.9	13	5.3	6.4
40–49	7	−1.9	3.9	13	−1.8	2.4	13	14.3	16.0	13	1.6	4.2	6	5.0	11.2	12	8.3	14.0	12	−4.5	15.6	12	−17.8	19.3	13	12.5	12.5	13	12.2	12.5
50–59	14	0.4	6.9	19	−1.5	4.0	19	11.1	11.1	19	2.1	4.6	4	2.9	4.4	15	6.5	10.5	18	−7.8	22.3	18	−21.7	21.7	14	5.9	7.9	14	1.2	6.5
60–69	28	−5.6	5.9	37	−3.7	5.2	37	5.5	5.6	37	1.7	6.5	4	−4.3	7.3	28	−0.8	6.0	32	−5.3	10.9	32	−29.6	29.6	29	5.9	7.9	29	0.8	7.8
70–79	39	−14.2	14.2	55	−11.1	11.1	55	−3.5	4.4	55	1.9	6.6	13	−11.8	11.9	44	−6.5	6.8	35	−11.1	17.9	35	−37.4	37.4	43	−1.0	4.1	43	−6.1	8.3
80–89	21	−23.6	23.6	28	−19.7	19.7	28	−12.3	12.3	27	−3.5	4.3	4	−21.7	21.7	21	−14.8	14.8	23	−22.4	22.4	23	−50.0	50.0	24	−7.1	7.3	24	−9.8	11.4
90+	6	−30.1	30.1	8	−27.0	27.0	8	−19.8	19.8	8	−9.0	9.0	2	−24.0	24.0	11	−23.5	23.5	6	−29.5	29.5	6	−55.2	55.2	10	−13.3	13.3	10	−13.7	13.7
TOT	130	−10.3	12.5	184	−8.1	9.6	184	1.1	9.1	182	0.9	5.8	38	−7.1	10.8	147	−3.5	10.5	144	−9.9	18.2	144	−30.7	31.4	152	2.6	8.6	152	−2.2	9.0
**Females**																														
20–29	3	−2.1	2.1	6	2.5	2.8	6	8.5	8.5	6	4.7	6.0	3	−2.4	3.5	4	−0.8	1.8	5	7.5	11.8	5	−1.4	1.8	0	‐	‐	0	‐	‐
30–39	1	−8.0	8.0	1	3.0	3.0	1	20.9	20.9	1	1.0	1.0	0	‐	‐	0	‐	‐	1	−13.7	13.7	1	−17.0	17.0	1	10.3	10.3	1	7.6	7.6
40–49	8	3.5	4.9	10	0.2	5.0	10	12.2	13.0	10	−0.2	3.2	5	−0.8	4.9	9	4.6	8.3	9	−3.1	10.3	9	−21.1	21.1	9	14.3	14.3	9	10.5	10.5
50–59	7	−0.2	4.3	10	1.9	5.3	10	13.6	13.6	10	3.7	6.5	2	−17.0	17.0	9	−2.4	8.7	7	−11.7	14.3	7	−29.7	29.7	10	5.0	8.2	10	0.3	5.6
60–69	10	−11.9	11.9	25	−4.9	5.4	25	4.0	4.3	25	0.5	8.4	3	−19.1	19.1	19	−4.9	7.8	18	−18.8	21.1	18	−41.0	41.0	20	2.6	5.3	20	−2.4	4.4
70–79	25	−15.2	15.2	50	−11.1	11.1	50	−3.2	3.5	47	1.7	5.8	3	−13.1	16.0	35	−10.3	10.9	37	−28.6	29.2	37	−46.4	46.4	34	0.1	5.3	34	−4.7	7.5
80–89	35	−25.4	25.4	62	−20.1	20.1	62	−12.7	12.7	61	−4.0	4.1	7	−23.8	23.8	47	−16.9	16.9	35	−35.3	35.3	35	−56.3	56.3	44	−5.6	6.5	44	−8.3	10.5
90+	14	−33.7	33.7	20	−29.2	29.2	20	−22.2	22.2	19	−11.2	11.2	3	−30.1	30.1	12	−22.6	22.6	12	−42.0	42.0	12	−67.7	67.7	14	−9.4	9.4	14	−12.8	12.8
TOT	103	−17.9	18.9	184	−13.4	14.4	184	−5.2	10.0	179	−1.7	6.0	26	−15.8	17.1	135	−11.1	13.0	124	−26.0	28.0	124	−45.6	45.7	132	−1.0	7.0	132	−4.9	8.6

Abbreviations: cs, composite score approach of FP15; *n*, number of individuals tested; re, regression equation approach of the FP15.

**TABLE 8:** Descriptive statistics of the Rougé‐Maillart (2009) method (A) and the probability of belonging to an age group (years) according to score category in our sample (B).

(A) Descriptive statistics											
Group	CS	Males					Females				
		*n*	Mean	SD	Median	Range	*n*	Mean	SD	Median	Range
I	7–10	–	–	–	–	–	1	24	–	24	24
II	11–14	12	28.1	4.1	28.5	20–36	5	23.6	2.4	23	21–27
III	15–18	13	37.5	5.9	38	30–49	7	42.3	2.6	42	39–47
IV	19–22	21	48.4	6.7	49	31–63	8	51.1	6.2	50	42–60
V	23–26	44	64.6	7.1	62	52–86	30	66.1	7.6	68	52–83
VI	27–30	72	76	7.5	76	51–93	89	79	6.8	79	56–94
VII	31–32	20	84.4	6.9	85	71–95	39	88.8	6.2	88	72–104

(B) Probability for each age group per age category							
Age	Overall score category						
group	I	II	III	IV	V	VI	VII
15–24	**1.000**	0.294	0.000	0.000	0.000	0.000	0.000
25–34	0.000	**0.647**	0.150	0.034	0.000	0.000	0.000
35–44	0.000	0.059	**0.750**	0.172	0.000	0.000	0.000
45–54	0.000	0.000	0.100	**0.621**	0.041	0.006	0.000
55–64	0.000	0.000	0.000	0.172	**0.459**	0.006	0.000
65–74	0.000	0.000	0.000	0.000	**0.432**	0.267	0.068
75–84	0.000	0.000	0.000	0.000	0.054	**0.540**	0.153
>84	0.000	0.000	0.000	0.000	0.014	0.180	**0.780**

*Notes:* Bold and dark grey backgrounds indicate the most probable age groups per overall score category. Lighter grey backgrounds indicate still acceptable age groups for a given score category.

Abbreviations: CS, Composite Score; *n*, numbers of individuals; SD, standard deviation.

It is important to remember that few individuals were in the first four score groups, so expanding the sample for younger age ranges would improve the interpretation.

For KU99, results were also assessed for the three distinct features described within the method, as summarized in Table 9. The tubercle exhibited the highest percentage of correctness (C%), reaching 98% in the female sample.

**TABLE 9:** Accuracy results for the three features evaluated separately of the Kunos 1999 method.

	Costal face					Tubercle facet					Rib head				
	*n*	C%	O%	B	I	*n*	C%	O%	B	I	*n*	C%	O%	B	I
Males	134	77.6%	22.4%	−7.9	12.3	104	85.6%	14.4%	−12.4	14.7	103	76.7%	23.3%	−7.2	12.3
Females	119	91.6%	8.4%	−17.6	18.3	102	98.0%	2.0%	−19.2	19.2	102	84.3%	15.7%	−11.5	13.7

Abbreviations: B, bias; C%, percentage of individuals correctly classified; I, inaccuracy; *n*, number of individuals tested; O%, percentage of individuals overestimates.

## 4 DISCUSSION

The study aimed to test various macroscopic methods for age‐at‐death estimation on a contemporary Italian sample to assess their applicability, reliability, and validity.

### 4.1 Applicability

Applicability was evaluated based on the preservation of relevant skeletal features. All individuals exhibited similar taphonomic characteristics. Overall, skeletal remains showed good preservation, with variation across skeletal regions. Specifically, the iliac portion of the innominate bone exhibited an excellent rate of preservation, allowing an extensive application of methods on the auricular surface (92% for LO85 and BC02). The acetabular portion of the innominate bone was also very well preserved, second only to the auricular surface, permitting application of RM09 in 90.2% of individuals. This result aligns with literature reports from various contexts, which emphasize that the os coxae, particularly the ilium, has a higher survival rate compared to other skeletal elements [68]. According to Waldron [68], the dense structure of the innominate contributes to its greater resistance to taphonomic processes, making it a key element for age estimation methods on skeletal remains.

Preservation results of the pubic symphysis were more mitigated, with SB90 applicable only in 58.3% of the sample. The lower preservation rate of the pubic portion compared to other anatomical areas of the innominate bone is evident in other contexts as well: preserved in two out of three cases in archaeological settings [68] often damaged by scavenger bites in open burial contexts [9].

The medial end of the clavicle was preserved in 71% of individuals, indicating a notable degree of resistance to taphonomic alterations. Palatine sutures were preserved well enough for assessment in almost 70% of individuals in the sample. This result contrasts with a previous study in the same cemetery context, where observability was only 26.2% of the sample [9].

The fourth rib provided the worst results with IS84‐85 applicable in only 16% of individuals. The bones of the rib cage are extremely fragile and highly susceptible to fractures and alterations caused by soil or taphonomic impacts in burial contexts [9]. Additionally, the recovery of disarticulated and fragmented rib remains complicates the identification of the fourth rib. Although the assessment of the fourth rib may be easier by exploiting its accessibility during autopsy on a fresh cadaver or by using medical imaging, such as X‐rays or 3D CT scans when maceration cannot be performed, the preservation and identification of the sternal end of the fourth rib in burial contexts remain challenging. This negatively affects the applicability of IS84‐85 in cemetery/archaeological settings. The applicability of the IS84‐85 method could be improved if it could be accurately extended to other ribs. Yoder et al. [34] found significant phase distribution differences only between the second and fourth ribs, suggesting that the method may also be applicable to other ribs (III, V–XI). However, the authors themselves emphasize the need for caution when applying the method to additional ribs due to limitations in statistical significance and recommend a composite score approach. Further studies in this area could enhance the accuracy of the method for ribs other than the fourth, thereby increasing its applicability across different contexts. In contrast, the first rib was preserved and identifiable in 70% of the individuals examined, confirming that it is more easily recognizable and less prone to taphonomic alterations than other ribs [35].

### 4.2 Intra‐ and inter‐observer reliability

Apart from methods assessing palatine sutures, the analyzed approaches proved reliable, demonstrating high intra‐ and inter‐observer agreement ranging from “substantial” to “perfect”. Overall, although no major differences emerged between observations or among observers, minor discrepancies suggest that techniques employing a narrower categorical scale tend to achieve higher intra‐ and inter‐observer agreement. This observation aligns with previous studies [e.g., 69]. For example, it is interesting to compare LO85, using eight categories, with BC02, which also assesses the auricular surface of the ilium but employs a composite score based on five variables using three‐ or five‐point scales: the scoring system of BC02 was introduced to reduce subjectivity in the staging system of the original LO85, as each feature is evaluated individually using ordinal scores, yet the introduction of multiple variables increased the potential for intra‐ and inter‐observer disagreement in the final composite score. This highlights the fine balance between repeatability and predictive accuracy, as increasing repeatability through score simplification may also lead to a loss of precision in age estimation.

In contrast, palatine suture obliteration did not show strong intra‐ or inter‐observer agreement, indicating high subjectivity, with “poor” or “slight” agreement reported for certain suture segments. Even Beauthier himself noted that different observers could arrive at varying evaluations due to the difficulty in assessing palatine suture obliteration [43]. Furthermore, percentage obliteration assessment appeared highly subjective and depended significantly on the observer's experience with the method. Since this seemingly subjective assessment must be repeated across 15 palatine suture subportions, substantial inter‐observer disagreement is introduced. The use of regression equations proved more susceptible to inter‐observer disagreement. In fact, the final estimate was never consistent in any of the inter‐observer analyses, with an average disagreement of 13 years. The intra‐observer analysis showed better agreement, with an average difference of only 2.2 years. Therefore, results show BE10 to be highly subjective, and the application of its regression equations can lead to inter‐observer differences greater than 10 years.

Validation tests of KU99 reported descriptions challenging to interpret and particularly subjective [38]. Additionally, combining the information of five features across three different regions, for a total of 15 parameters, is quite complex to manage. Conversely, Kunos et al. [35] consistently reported reliability among observers. However, in their study, Observer 1 was the developer of the technique, Observer 2 learned the technique by examining the first ribs of known age with detailed notes, and Observer 3 learned directly from Observer 1. Moreover, given that the inter‐observer analysis conducted by Kunos et al. is based only on mean differences between observations, the reported averages may mask significant individual variation. The results of the present study support good method repeatability, with “almost perfect” intra‐ and inter‐observer agreement for costal face assessment and “substantial” agreement for the tubercle and costal head assessments. However, as in the original study, Observer 1 learned the method directly from Observer 2, and both observers have substantial experience applying the technique. Yet the technique is challenging for untrained observers, impacting repeatability among observers with varying levels of experience [38].

### 4.3 Accuracy

Given the demographic distribution of the sample, several considerations had to be implemented. First, as the age distribution of the sample was not homogeneous, with 77.5% of individuals with a chronological age‐at‐death of over 60 years, interpretations per age group were recommended. Second, age distribution between male and female samples was significantly different (*p* < 0.01), with the female sample being comparatively older; thus, a separate analysis had to be conducted for each sex. Furthermore, the fact that biological age increasingly diverges from chronological age over time due to the accumulation of extrinsic factors [3] influenced our results, with methods showing a generally lower correlation for the older female sample.

#### 4.3.1 Lovejoy 1985

Considering one standard deviation, LO85 had the best rate of correct classification, 79.3% for males and 80.3% for females. However, the method's final stage provides a general age estimate of “over 60 years,” making it less precise for estimating age in older individuals. As shown in Table 6, accuracy is nuanced around this threshold: over 60, the method correctly classifies individuals in 88.8% of cases, whereas under 60, this percentage drops considerably to 56.6%. The method thus effectively distinguishes between individuals over and under 60 years of age. Indeed, only 11.1% of individuals over 60 were incorrectly scored, none of them under 52 years. However, the method is not able to provide age ranges over 60 years. Hens and Godde [24] have shown that a Bayesian approach can be used to define narrower age ranges for older adults, partially mitigating the limitations of the original method. This issue is particularly relevant given the ongoing increase in life expectancy observed today, increasing the likelihood of encountering human remains of unknown individuals with ages‐at‐death over 60 [70].

In contrast, among those aged 20–40, for both sexes, the method tended to slightly overestimate the actual age of individuals, with an average inaccuracy varying from 3 to 4 years. This result is consistent with what has been found in the literature, including a study on an Italian population that reports an overestimation in individuals under 40 years, with an inaccuracy ranging from 1 to 4 years [62]. Between 40 and 60 years, the method consistently underestimated age in males and slightly overestimated age in females. The female sample's result diverged from other validation tests, reporting an underestimation for both sexes [51, 62, 71]. Inaccuracy increased slightly compared to younger age groups, averaging around 5 years for both sexes, though lower than reported in other populations [51, 62, 71, 72].

Despite an inaccuracy of 5 years or less for individuals under 60, the method correctly classified just over half of these individuals. This is related to the overly narrow age range (5 years) assigned to the first six stages of the method. This finding aligns with the literature, indicating that a final interval of 5 years is too narrow to best represent observable variability [21].

The method requires a statistical modeling of the stages to obtain less precise but more valid age ranges. Hens and Godde [73] demonstrated significant improvements to the method by using a Bayesian statistical approach on a combined population of two samples, one American and one Portuguese. Expanding the current sample of younger age groups could allow for the application of a similar approach, yielding significant advances applicable to the Italian population as well.

#### 4.3.2 Buckberry and Chamberlain 2002

Both BC02 and LO85 reported similar validity results and a strong positive association with age, with the revised method being slightly better correlated for females and the original method slightly better for males. Correlation coefficients (*R* = 0.868 for males and *R* = 0.845 for females) were higher than those reported for the Spitalfields sample in the original validation test, *R* = 0.624 for males and *R* = 0.626 for females, and other validation tests in different populations [23, 63, 74]. Macroporosity was found to be the characteristic relatively least correlated with age‐at‐death (though still showing good correlation), in complete contrast to the original result, which identified it as the most strongly correlated with chronological age‐at‐death [22]. However, this aligns with a previous test conducted on a different Italian population [63]. The original validation test shows a lower correlation coefficient for apical activity, whereas in the present study, all features were strongly correlated with age‐at‐death, with surface texture and apical activity being the most strongly correlated features. In general, it is noted that different validation tests report varying results for the correlation coefficients of individual characters assessed by the method [74, 75]. This variation, in addition to inter‐population differences, can be influenced by the different age distributions of the samples and the individual experience of the observers with the method.

In general, BC02 strongly overestimated individuals under 60 years, with an inaccuracy generally exceeding 10 years. For both sexes, the method overestimated the result up to the 60–69 age range, although in this case, the inaccuracy was considerably lower. Indeed, the method performed better for individuals within the 60–79 age range, with inaccuracy levels kept under 6 years and a classification rate of 100% for both sexes. Above 70 years, the method significantly underestimated chronological age‐at‐death, since the last auricular surface stage gives an estimated age of 73 years. This is supported by other validation tests [23, 76, 77], including on a different Italian population [63]. This trend is possibly due to an effect of age mimicry with the original sample, which, as indicated by the authors themselves, included an overrepresentation of older adults, causing the overestimation of younger individuals in independent samples.

BC02 exhibited higher levels of bias and inaccuracy for individuals under 60 years compared to the original LO85; on the other hand, the revised method performs better for age ranges between 60 and 80 years and reduces inaccuracy for those over 80. Several authors have compared the two methods using the same sample [23, 30, 63, 73, 74, 78], concluding that the revised method is more accurate for estimating the age of older individuals, while it tends to more strongly overestimate those under 50 years old. This conclusion is supported by our data. Despite the lower inaccuracy reported for older individuals, the age ranges of the revised method are too broad and not satisfactory. For instance, stage 6 spans from 42 to 94 years, and stage 7 from 52 to 104 years (Table S1).

#### 4.3.3 Suchey‐Brooks 1990

In general, the aging stages of the auricular surface showed a stronger correlation with age‐at‐death and better validity compared to the pubic symphysis. Despite this, the stages indicated by SB90 still show a close association with age‐at‐death, consistent with what is reported in the literature [18, 36, 79, 80], and the method has proven valid for estimating the age‐at‐death of younger individuals. The low percentage of correct classifications found for the method (53.1% for males and 31.1% for females), considering one standard deviation, is strongly influenced by the lack of maximum age in the last stage (like LO85) but rather an age range up to 72–73 years, thereby automatically excluding most of individuals in the sample. The method did not prove useful for estimating the age of individuals over 60, who were strongly underestimated for both sexes, with inaccuracies progressively increasing with age from 12 to more than 30 years. Yet, it exhibited better accuracy for individuals under 60, with a slight overestimation for younger males and a slight underestimation for younger females, but with levels of inaccuracy contained within 7 years and a correct classification rate of 100% using two standard deviations. According to the original authors, the method is more accurate for young adults (up to 40 years) in a modern North American sample. Over this threshold, they found significant individual variability, which led to very wide age distributions. This result was also observed in subsequent validation tests in different population contexts [16, 51, 62, 77, 79, 80]. Despite our limited number of younger individuals, our result seemed to confirm the findings described in the literature, though with a significantly smaller bias and inaccuracies for the age ranges between 40 and 70 years, potentially from a population effect. Since the ventral rampart of the pubic symphysis fuses by 35 years [81], after this age, only degenerative changes are observed. These changes are much harder to interpret and tend to vary greatly due to factors such as lifestyle, environment, and population genetics [71]. Therefore, in our sample, there could be less variability with older individuals being more strongly correlated with the last two aging stages of the pubic symphysis. Specifically, the last stage (stage 6) was never assigned to individuals younger than 49 years, allowing a distinction between those over and under 50 for both sexes (Table S3).

#### 4.3.4 Iscan 1984–1985

A positive correlation was observed with age, consistent with the literature [82, 83], although slightly lower compared to the auricular surface and pubic symphysis. In addition to the strong applicability limitations previously described, the method exhibited lower accuracies than previous methods. The actual age of the individuals examined was within the estimated range for only 55% of males and 26.9% of females. The method proved valid for individuals under 40, with extremely low bias and inaccuracies, though the sample size was very small limiting its validation. Above 40 years, the method strongly underestimated female ages, with inaccuracies of 15–20 years for the middle age groups, overestimated male aged 40–60, with inaccuracies ranging from 4 to 11 years. Over 60 years, male ages were underestimated, with progressively larger inaccuracies. Although some validation tests found excellent overall accuracy for the method, similar to the original method [84, 85, 86, 87], other studies concur on its validity for individuals under 40 and age underestimation after 40, with progressively greater inaccuracies [82, 83, 88]. Literature shows that the process of aging at the sternal end of the rib differs between populations, impacting age estimation [82, 89]. For the Italian population, Cappella et al. (2017) [9] also reported low validity, with a 54% success rate. However, similar to the present study, this result was influenced by a limited sample due to the poor applicability of the method. An increase in sample sizes could provide more valid interpretations in the Italian population.

#### 4.3.5 Kunos 1999

Similar to the fourth rib, KU99 showed a good correlation with age, consistent with Kurki [37]. One noteworthy point of concern is the uncertainty regarding the application procedure of the method. Indeed, the method lacks clear guidelines on how to determine age ranges or how to combine the evaluation of the three areas of interest for a final estimate, causing inconsistent results and risking improper applications. As a result, each practitioner may interpret and apply the method differently, compromising repeatability. This issue is also noted in two validation tests [37, 38], in which the method was applied differently. Kurki [37], who tested the method on a small sample from the Grant Collection, indicated a minimum age corresponding to the “oldest” morphology observed, which he used to calculate bias and inaccuracies, an approach we shared. Alternatively, Schmitt and Murail [38] observed morphological changes in the three anatomical areas and then assigned a decadal range, considering the costal face as the main criterion. Our results showed that, in 32% of males and 15.6% of females, the chronological age was lower than the “minimum age” indicated by the final estimate of the method. A closer look at the results revealed substantial validity issues with this approach. In fact, the technique indicated an age up to 76 years for the costal face, 75 years for the costal head, and 73 years for the costal tubercle. This means that individuals older than these ages were automatically classified correctly using this approach, regardless of the observed morphology. If only individuals under 60 years of age are considered, the accuracy of the methodology decreases considerably: 45.5% of females indicated a minimum age older than the actual age, with an average error of 9 years, and 76.7% of males were overestimated, with an average of 13 years. This is consistent with findings from other validation tests, showing an overestimation of individuals up to 60 years, while being valid for individuals over 60 [37, 38]. KU99 can still be considered accurate within a multifactorial approach, useful for defining the lower limit of a range, though it may lead to significant overestimation of age in young‐ and middle‐aged adults.

In the female sample, the tubercle showed an excellent correlation with age‐at‐death (*R* = 0.800), and only two individuals (2.0%) were overestimated (under 4 years). The costal tubercle appears to be a valid indicator for estimating a minimum age‐at‐death in females. In contrast, the costal face and rib head for both sexes and the tubercle for males can lead to overestimation by over 30 years.

Thus, despite its good correlation with age and high preservation rate, KU99 needs to be revised, standardized, and tested in different population contexts to be safely applied.

#### 4.3.6 Palatine sutures

Both MA91 and BE10 exhibited mediocre results for estimating age‐at‐death, with a poor correlation with age. While in the male sample this correlation was low but still statistically significant, in the female sample, the degree of obliteration of the IN and AMP sutures was not significantly correlated with age‐at‐death. Both methods resulted in a substantial underestimation of individuals across all age groups, with particularly high levels of inaccuracy and low percentages of correct classification. Specifically, correct classification with BE10 occurred in only one‐third of males and 7% of females. The method showed a severe underestimation of chronological age‐at‐death, with inaccuracies ranging from 11 to 42 years across all age groups for both sexes. The author of the method himself stated that “palatine sutures generally do not estimate age‐at‐death better than cranial vault sutures” [43, p.153], but also later asserted that “palatine sutures observation contributes additional information for age estimation, especially in older and very old individuals, where other methods lose their effectiveness” [43, p.153]. Cappella et al. [9] tested it on a sample from the CAL Milano Cemetery Skeletal Collection, reporting its utility for individuals aged 60–80 but with an underestimation of those over 80. However, this study was based on a limited sample, with only 38 individuals. The present study, with a larger sample and all age groups represented, shows critical validity for both younger and older individuals, and, contrary to previous study [9], underestimation of individuals over 60 with inaccuracies of over 20 years.

MA91 produced even more mediocre results in terms of validity: only 25% of males and 12.9% of females were correctly classified, with an average inaccuracy of 31.4 years for males and 45.7 years for females. In particular, the validity was strongly limited for females, for whom no statistically significant correlation was found with age (*R* = 0.157; *p* = 0.082). Lower correlation with age for females has been reported in the literature [43, 90, 91]. Particularly problematic is the lack of correlation for the IN suture in females (*R* = 0.041; *p* = 0.633), as this pertains to the first step of the method. Low rates of correct classification have been reported for this method [42, 44, 90, 91]. However, while, in other studies, the method tended to overestimate the age of younger individuals and underestimate that of older ones [87], in our study, it consistently underestimated age‐at‐death, regardless of the individuals' actual age. Recent advancements, such as the Bayesian approach proposed by Hens and Godde [44], suggest that alternative statistical methods may help refine age estimates by reducing bias and providing narrower prediction intervals. Their findings further support the notion that while palatal sutures alone may not be accurate, they can still contribute additional information in age estimation, particularly for older individuals, when integrated with more robust statistical frameworks.

#### 4.3.7 Rougé‐Maillart (2009)

RM09 exhibited the best accuracy across age groups, with biases close to zero and limited inaccuracies across all age ranges and both sexes. Correlation with age was almost perfect for both sexes, as observed in the Colombian (*R* = 0.860) [77] and American populations (*R* = 0.77) [92]. This highlights the method's great potential for age estimation. Unlike other methods, RM09 showed an excellent balance, without a clear trend toward either over‐ or under‐estimation of individuals. The only trend noted was that young adults (20–29 years) tended to be overestimated by an average of 4–6 years. However, the sample for this age group is extremely limited in number, and the levels of bias and inaccuracy are still more contained than those of BC02. Individuals 90+ were, unsurprisingly, underestimated in age. However, the fact that this method allows for age estimation up to 84 years significantly reduces the underestimation of older individuals. For the 80–89 age group, the method reported an inaccuracy of only 4 years for both sexes, and for the 90+ category, the underestimation was only of 9–11 years, whereas previous methods generally showed underestimations with inaccuracies of over 20 years. This indicates that the method is potentially highly accurate for the entire span of adulthood.

Despite the limited bias and inaccuracies, the final age range indicated by the method included the actual age of individuals in only about 60% of the sample. This outcome highlights an issue in the method's association between composite score and age range. The method groups the final scores into seven score categories, with each score category associated with a decade‐long age interval (the last group being associated with >84 years) where it is most likely that the group belongs, calculated using a Bayesian approach with posterior probabilities. However, seven score groups are associated with eight age categories. Consequently, by considering only the most probable category, one age class is automatically excluded. Specifically, the 65–74 age range (since Group V is associated with the 55–64 range, and Group VI with the 75–84 range), which represents 22% of the sample. Additionally, 10‐year age intervals are still too narrow, particularly for older individuals. Nonetheless, RM09 allows for more accurate estimation of older individuals, where other discussed methods fail, with the potential to distinguish between individuals above and below 84 years. Despite a slight overlap between age classes, considerations in our sample indicate that narrower age ranges even for older individuals are possible using this method. Testing with an expanded sample for younger age ranges and revising the scores for each criterion in the method may help improve it and reduce overlap among different age classes. Currently, the method does not yet enjoy high regard in practical application, so validation tests are limited. Cappella et al. [9] tested the method on a small sample of individuals over 60, reporting that the technique showed good potential in estimating individuals over 60. Rivera‐Sandoval et al. [77], on a Colombian sample, reported good accuracy, with a tendency to underestimate older individuals. Interestingly, they found that BC02 proved more accurate than RM09 in older individuals [77], dissimilarly from our study. This result highlights possible inter‐population specificity in acetabular degeneration progression, in accordance with the literature [93, 94, 95].

Other studies have highlighted that methods based on the acetabulum tend to underestimate age [96]. However, it has been found that these methods are more suitable for people over 40 [26, 96, 97], and even more so for those over 60 [9]. Moreover, this study shows that all observed traits of the acetabulum correlate with age‐at‐death similar to auricular surface characteristics and that combining the acetabulum with the auricular surface in the RM09 method does not lead to underestimation but instead confirms its potential for older age estimation.

#### 4.3.8 Falys and Prangle (2015)

FP15, published recently, has drawn interest from the scientific community due to promising results for estimating age‐at‐death of older individuals. This method has already been tested in five different populations, with mixed results. Madentzoglou et al. [50] conducted tests on both a Greek and a Thai sample, reporting lower accuracies than those originally indicated for the regression equation (43.3%–56.7% for the Thai and 61.2%–63.2% for the Greek), with a pronounced tendency to significantly overestimate individuals younger than 40 and underestimate those over 80. Furthermore, a significant correlation with age was found only for topography in Thai females, while none was present in males, unlike the Greek sample, which showed a strong correlation with age‐at‐death. The authors attribute these findings to differences potentially related to the sample itself, ethnicity, nutrition, or physical activity [50]. In an African American sample from the Hamann‐Todd Collection [98], accuracies of only 38.4% for the composite score approach and 78.6% for logistic regression were reported, both lower than the original 96% from Falys and Prangle. Price [99] examined European American individuals in the McCormick Collection and the William M. Bass Donated Skeletal Collection, analyzing the largest sample to date (1510 individuals, ages at death from 20 to 101). This study also failed to find the same accuracy percentages as the original method for both logistic regression (47.6% for males and 57.4% for females) and the composite score approach (males = 65.9%; females = 58.8%). The method was also tested on a 18th–19th centuries Dutch population [52], reporting an accuracy of 87% in the composite score approach, and on cremated adult remains [53], demonstrating a strong concordance with age.

Our correlation coefficient results indicated that all three observed traits correlate closely with age‐at‐death, with composite scores showing an association with age comparable to that found for the degeneration of the auricular surface and the pubic symphysis. Furthermore, porosity proved to be the feature most strongly correlated with age‐at‐death in both sexes, dissimilarly with the literature. All other validation tests indicate that topography is the trait most closely associated with age [49, 50, 98, 99], so much so that topography alone was considered an accurate age indicator [100]. This finding is not confirmed in the present study, where all three traits showed a mostly equally strong association with age‐at‐death.

As for the accuracy of the method, the present study provided results lower than the original but still superior to those of other validation tests: 59.2% correct classification for males and 60.6% for females with the composite score approach. The 95% confidence interval was not met, with 87.5% correctly classified males and 84.1% correctly classified females. For logistic regression, results were similar for males and slightly better for females, with the 95% confidence interval closely met (94.7%). The method lost precision with just one standard deviation, as age intervals exceeded 30 years. Furthermore, both approaches showed a strong overestimation of individuals under 50 and an underestimation of those over 80, consistent with the literature [50, 99]. Conversely, for ages between 50 and 70, bias was closer to zero, and inaccuracies were contained within 8 years.

The validity results observed reflect certain intrinsic issues in the methodology. First, the method was designed to estimate age‐at‐death of individuals over 40 years, excluding younger individuals who may already exhibit complete fusion of the sternal end of the clavicle. Various studies suggest that this may occur as early as age 25 [46, 48, 101]. The omission of younger individuals in the method necessarily leads to an overestimation of younger age ranges. Furthermore, regarding the composite score approach, the oldest female age group showed a severe issue regarding the indicated age range: in the original study, composite scores above 13 were rarely assigned to the female sample, leading to an age range of 86–88 years (within one standard deviation) and 85–89 years (within a 95% confidence interval) for the final age group. These narrow age ranges are extremely limited in capturing observable variability, particularly for older adults. In our sample, 20% of the females were estimated in the last age group, negatively affecting the accuracy results for the female sample (this reason also accounts for the lower accuracy observed in the composite score approach compared to logistic regression).

Nonetheless, the technique, particularly the composite score approach, exhibited inaccuracy levels second only to RM09. For example, from the descriptive statistics (Table S6), two notable insights can be drawn: (1) within age class V, all males were at least 75 years old (with 71% over 80), and all females were at least 70 years old (with 81% over 80); (2) within age class IV, 75% of the male individuals were over 70 years old, and this percentage rose to 97% in the female sample.

Thus, although the method is less accurate than suggested by the original authors, it remains reliable for estimating older ages, in particular for individuals over 70. In contrast to previously discussed methods, and similar to RM09, FP15 demonstrates good validity in distinguishing individuals over 70 and over 80 years old.

The findings of this research provide valuable insights into how and when different methodologies should be applied, as well as which results should be considered more accurate. In age‐at‐death estimation, it is well established that different skeletal indicators may vary in accuracy for different age groups. As a result, researchers have considered the potential benefits of combining multiple skeletal traits to enhance accuracy and precision. Traditionally, the way information from different methods is integrated has been left to the subjective decision of the observer, who may choose to prioritize certain estimates based on personal experience. However, an alternative approach is provided by transition analysis, which systematically combines multiple indicators to enhance accuracy while addressing the bias of age mimicry. While this study does not test such an approach, future research could evaluate its applicability and predictive accuracy in comparison to traditional methods.

Lastly, we acknowledge that the choice of assessing only one side of the skeletal indicators could be a limitation, as it may not account for potential asymmetries that could affect age‐at‐death estimation. In future research, it would be beneficial to test both sides of the body to better understand the role of asymmetry in age estimation. This approach could provide valuable insights into how bilateral differences may influence the accuracy and reliability of age‐at‐death assessments when applying these methods.

## 5 CONCLUSION

The age‐at‐death estimation methods tested demonstrated variable accuracy depending on the age group considered. For example, methods like SB90 are particularly effective for estimating ages in younger individuals, whereas for older individuals, RM09 and FP15 show lower bias and inaccuracy. However, certain limitations in these methods—such as limited precision and issues with classification in the oldest age ranges—indicate that no single method currently provides a fully satisfactory solution for accurately estimating the ages of older adult individuals. Although RM09 and FP15 showed great potential, with strong correlations with age‐at‐death, correct classifications provided unsatisfactory results, showing opportunity for improvement. The limitations in age‐at‐death estimation for older individuals have significant repercussions in both forensic and bioarchaeological anthropology. In forensic contexts, imprecise age estimates complicate the identification of older adults, whose remains may belong to vulnerable or missing populations, including those who may lack accessible identification records. This inaccuracy can hinder efforts to narrow down search parameters or to confirm the identity of unknown people, especially as elder populations continue to grow globally and as forensic cases involving older individuals become more frequent. Similarly, in bioarchaeology, broad age‐at‐death categories like “50+” hinder the ability to accurately interpret demographic patterns, as they mask age‐related mortality trends and obscure our understanding of health, status, and aging within past populations. Narrowing age ranges for older adults could greatly enhance the resolution of age‐related demographic profiles and clarify distinctions between life expectancies in different historical periods and cultural settings.

Given the continuous rise in life expectancy, the need to address these methodological gaps is increasingly urgent. The growing likelihood of encountering older adults’ remains calls for refined age‐at‐death estimation techniques that can better accommodate the age diversity seen in contemporary and ancient populations alike. Improved methods would provide more accurate assessments of age‐at‐death for older individuals, not only meeting the demands of current forensic and bioarchaeological work but also offering a more nuanced understanding of aging, mortality, and population structures across human history.

## CONFLICT OF INTEREST STATEMENT

The authors declare no conflict of interest.

## Supporting information


Table S1:

## Data Availability

The data that support the findings of this study are available from the corresponding author [PM] upon reasonable request.
