# Supplementary material for: Applicability, reliability, and accuracy of age‐at‐death estimation methods on a contemporary Italian population
Source: J Forensic Sci. 2025 Mar 13;70(3):868–88. doi: 10.1111/1556-4029.70011 (PMC12046114; doi:10.1111/1556-4029.70011)
Supplement: Supplementary file 1 — Table S1: [file JFO-70-868-s001.docx]

**Supplementary Material**

TABLE S1 Descriptive statistics of the Buckberry and Chamberlain 2002 method.

| **Group** | **CS** | **males** | | | | | **females** | | | | |
| --- | --- | --- | --- | --- | --- | --- | --- | --- | --- | --- | --- |
|  |  | **n** | **mean** | **SD** | **median** | **range** | **n** | **mean** | **SD** | **median** | **range** |
| I | 5-6 | - | - | - | - | - | - | - | - | - | - |
| II | 7-8 | 4 | 26.8 | 6.7 | 25.5 | 20-36 | 4 | 23.8 | 2.5 | 23.5 | 21-27 |
| III | 9-10 | 11 | 32.1 | 6.9 | 29 | 24-49 | 3 | 29.7 | 10.8 | 25 | 22-42 |
| IV | 11-12 | 7 | 37 | 7.2 | 35 | 30-51 | 4 | 43.5 | 2.7 | 43 | 41-47 |
| V | 13-14 | 17 | 46.7 | 10.2 | 46 | 30-72 | 3 | 41.7 | 3.1 | 41 | 39-45 |
| VI | 15-16 | 37 | 61.7 | 10.4 | 61 | 44-86 | 26 | 64 | 11.6 | 62.5 | 42-94 |
| VII | 17-19 | 108 | 75.9 | 9.0 | 76 | 56-95 | 144 | 80.3 | 9.2 | 81 | 52-104 |

CS=Composite Score; n=numbers of individuals; SD=Standard Deviation

TABLE S2 Descriptive statistics of the Lovejoy 1985 method.

| **stage** | **males** | | | | | **females** | | | | |
| --- | --- | --- | --- | --- | --- | --- | --- | --- | --- | --- |
|  | **n** | **mean** | **SD** | **median** | **range** | **n** | **mean** | **SD** | **median** | **range** |
| **1** | - | - | - | - | - | 1 | 23 | - | 23 | 23 |
| **2** | 4 | 24.8 | 3.4 | 25.5 | 20-28 | 5 | 23.8 | 2.4 | 24 | 21-27 |
| **3** | 6 | 28.8 | 2.9 | 29 | 24-33 | - | - | - | - | - |
| **4** | 11 | 35.2 | 5.8 | 35 | 29-49 | 3 | 42.3 | 1.5 | 42 | 41-44 |
| **5** | 11 | 42.9 | 6.3 | 43 | 31-54 | 5 | 42.8 | 3.2 | 42 | 39-47 |
| **6** | 12 | 53.3 | 9.1 | 49 | 44-74 | 1 | 56 | - | 56 | 56 |
| **7** | 33 | 61.9 | 9.2 | 60 | 50-86 | 26 | 63.7 | 10.7 | 64.5 | 42-89 |
| **8** | 107 | 76.4 | 8.4 | 76 | 60-95 | 143 | 80.6 | 9.1 | 81 | 52-104 |

n=numbers of individuals; SD=Standard Deviation

TABLE S3 Descriptive statistics of the Suchey-Brooks 1990 method.

| **phase** | **males** | | | | | **females** | | | | |
| --- | --- | --- | --- | --- | --- | --- | --- | --- | --- | --- |
|  | **n** | **mean** | **SD** | **median** | **range** | **N°** | **mean** | **SD** | **median** | **range** |
| **1** | - | - | - | - | - | 2 | 21.5 | 0.7 | 21.5 | 21-22 |
| **2** | 2 | 25.5 | 0.7 | 25.5 | 25-26 | 1 | 27 | - | 27 | 27 |
| **3** | 2 | 27 | 4.2 | 27 | 24-30 | 1 | 39 | - | 39 | 39 |
| **4** | 9 | 39.9 | 9.5 | 39 | 28-60 | 2 | 41 | 0 | 41 | 41 |
| **5** | 20 | 49.9 | 14.8 | 50 | 29-86 | 16 | 58.8 | 13.7 | 58.5 | 42-83 |
| **6** | 97 | 73.6 | 10.1 | 75 | 50-95 | 81 | 80.4 | 10.5 | 82 | 49-101 |

n=numbers of individuals; SD=Standard Deviation

TABLE S4 Descriptive statistics of the Iscan 1984-1985 method.

| **phase** | **males** | | | | | **females** | | | | |
| --- | --- | --- | --- | --- | --- | --- | --- | --- | --- | --- |
|  | **n** | **mean** | **SD** | **median** | **range** | **n** | **mean** | **SD** | **median** | **range** |
| **0** | - | - | - | - | - | - | - | - | - | - |
| **1** | - | - | - | - | - | - | - | - | - | - |
| **2** | - | - | - | - | - | 1 | 22 | - | 22 | 22 |
| **3** | - | - | - | - | - | 2 | 24 | 4.2 | 24 | 21-27 |
| **4** | 4 | 34.3 | 8.5 | 30.5 | 29-47 | 1 | 41 | - | 41 | 41 |
| **5** | 2 | 39 | 0 | 39 | 39 | 6 | 53.8 | 11.1 | 57 | 40-68 |
| **6** | 9 | 58.4 | 14.5 | 51 | 44-86 | 10 | 73.6 | 18.1 | 77 | 42-94 |
| **7** | 11 | 67.8 | 14.1 | 71 | 43-83 | 3 | 83 | 4.4 | 81 | 80-88 |
| **8** | 12 | 77.7 | 11.6 | 77.5 | 57-101 | 3 | 84.7 | 11.4 | 88 | 72-94 |

n=numbers of individuals; SD=Standard Deviation

TABLE S5 Descriptive statistics of the Mann 1991 method.

| **Age group** | **males** | | | | | **females** | | | | |
| --- | --- | --- | --- | --- | --- | --- | --- | --- | --- | --- |
|  | **n** | **mean** | **SD** | **median** | **range** | **n** | **mean** | **SD** | **median** | **range** |
| **1** | 16 | 53.5 | 13.9 | 56 | 24-77 | 22 | 74.5 | 17.1 | 76 | 22-104 |
| **2** | 7 | 31.3 | 3.7 | 30 | 28-39 | 39 | 66.0 | 21.3 | 71 | 21-95 |
| **3** | 62 | 66.4 | 16.4 | 68 | 29-93 | 49 | 74.4 | 13.4 | 74 | 41-97 |
| **4** | 20 | 74.3 | 11.5 | 75.5 | 50-95 | - | - | - | - | - |
| **5** | 39 | 67.8 | 15.5 | 68 | 31-101 | 14 | 80 | 6.7 | 79.5 | 69-91 |

n=numbers of individuals; SD=Standard Deviation

TABLE S6 Descriptive statistics of the Falys and Prangle 2015 composite score approach.

| **Group** | **CS** | **males** | | | | | **females** | | | | |
| --- | --- | --- | --- | --- | --- | --- | --- | --- | --- | --- | --- |
|  |  | **n** | **mean** | **SD** | **median** | **range** | **n** | **mean** | **SD** | **median** | **range** |
| **I** | **5-6** | 20 | 34.7 | 7.5 | 34 | 24-54 | 9 | 47.7 | 8.3 | 42 | 39-60 |
| **II** | **7-8** | 18 | 49.8 | 8.9 | 50 | 30-68 | 26 | 62.8 | 11.8 | 64.5 | 40-87 |
| **III** | **9-10** | 26 | 63.7 | 9.1 | 66.5 | 44-78 | 37 | 74.4 | 8.9 | 73 | 56-97 |
| **IV** | **11-12** | 71 | 75.1 | 10.2 | 77 | 42-93 | 34 | 82.5 | 6.0 | 83 | 68-91 |
| **V** | **13-14** | 17 | 84.4 | 7.8 | 84 | 75-101 | 26 | 87.0 | 7.4 | 87.5 | 70-104 |

CS=Composite Score; n=numbers of individuals; SD=Standard Deviation
